# Supplementary material for: The Morphology Dependent Interaction between Silver Nanoparticles and Bovine Serum Albumin
Source: Materials (Basel). 2023 Aug 25;16(17):5821. doi: 10.3390/ma16175821 (PMC10488934; doi:10.3390/ma16175821)
Supplement: Supplementary file 1 [file materials-16-05821-s001.zip › materials-1990644-SI.pdf]

The Morphology Dependent Interaction between Silver Nanoparticles and Bovine Serum Albumin

Jingyi Zhang [1], Xianjun Fu [1], Changling Yan [2] and Gongke Wang [1,*]

[1] Henan Engineering Research Center of Design and Recycle for Advanced Electrochemical Energy Storage Materials, School of Materials Science and Engineering, Henan Normal University, Xinxiang 453007, China
[2] Key Laboratory of Green Chemical Media and Reactions, Ministry of Education, Collaborative Innovation Center of Henan Province for Green Manufacturing of Fine Chemicals, School of Chemistry and Chemical Engineering, Henan Normal University, Xinxiang 453007, China
* Correspondence: wanggongke@126.com

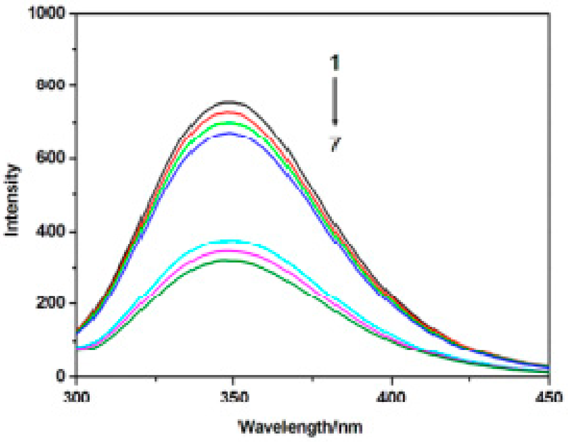

Figure S1. The effects of surface ligands of AgNPs on the fluorescence spectroscopy of BSA. From 1-7: they represent BSA, 5 μM), sodium citrate (1 mM), CTAB (1 mM), PVP (1 mM), spherical AgNPs (7×10^{-10} M), rod-shaped AgNPs (1.5×10^{-9} M) and triangular AgNPs (3.5×10^{-4} M).

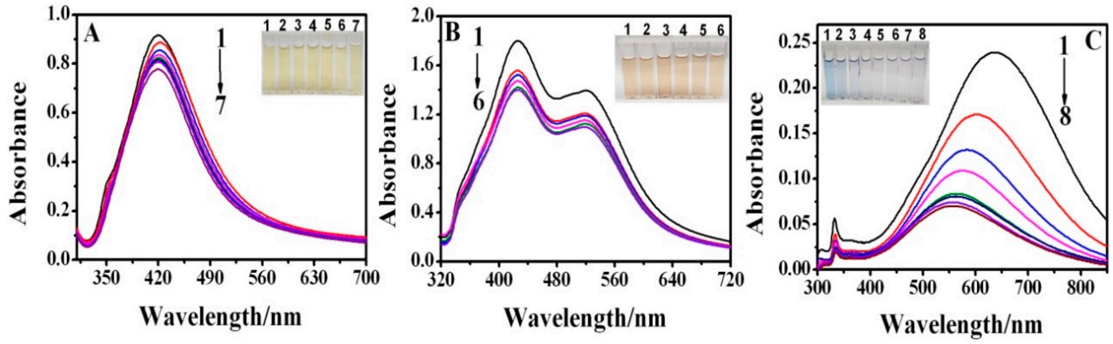

Figure S2. UV–visible spectrum of interaction of BSA with silver nanospheres (A), nanorods (B) and nanotriangles (C). From A to C, the concentrations of BSA are 0-2.4×10^{-5} M (A), the concentrations of BSA are 0-5×10^{-6} M (B), the concentrations of BSA are 0-7×10^{-5} M (C).

Table S1. Stern-Volmer quenching constants of the interaction between BSA and AgNPs with different morphologies.

Systems	K_{sv} ($L\cdot mol^{-1}$)	K_q ($L\cdot mol^{-1}\cdot s^{-1}$)	R^a
Nanospheres-BSA	$(6.56\pm0.28)\times10^{9}$	$(6.56\pm0.28)\times10^{17}$	0.9928
Nanorods-BSA	$(1.32\pm0.09)\times10^{9}$	$(1.32\pm0.09)\times10^{17}$	0.9714
Nanotriangles-BSA	$(5.04\pm0.36)\times10^{3}$	$(5.04\pm0.36)\times10^{11}$	0.9868

[a] R is the correlation coefficient.

Table S2. Conformation changes of the secondary structure of BSA with different morphologies of AgNPs, calculated by CDNN software.

Systems	Helix （%）	Antiparallel （%）	Parallel （%）	β-Tur （%）	Rndm.Coil （%）
BSA	58.4	2.9	6.4	12.8	21.1
Nanospheres-BSA	67.5	2.7	5.4	11.0	17.1
Nanorods-BSA	43.8	3.9	7.1	14.9	29.5
Nanotriangles-BSA	60.2	2.5	6.2	12.6	20.1
